# The Impact of High lncRNA Expression on Clinicopathological Characteristics and Prognosis of Endometrial Cancer Patients: A Meta‐Analysis

**DOI:** 10.1002/cam4.70755

**Published:** 2025-03-12

**Authors:** Xiaotong Zhao, Ziling Yang, Tianjiao Zheng, Mengyao Zeng, Xiaowen Lin, Huixin Chen, Weiqin Zheng, Sizheng Peng, Shibo Li, Tao Song, Yuhui Sun

**Affiliations:** ^1^ Department of Gynecology The First Affiliated Hospital of Harbin Medical University Harbin Heilongjiang China; ^2^ Department of Cardiology The First Affiliated Hospital of Harbin Medical University Harbin Heilongjiang China

**Keywords:** clinicopathological characteristics, endometrial carcinoma, long non‐coding RNA, meta‐analysis, prognosis

## Abstract

**Backgrounds:**

A growing number of systematic bioinformatics analyses were conducted to investigate the mechanism of interaction between long non‐coding RNA (lncRNA) and endometrial carcinoma (EC) to predict the prognosis. However, there is no evidence‐based evidence that abnormal lncRNA expression is strongly associated with the pathological characteristics and prognosis of EC patients. In this meta‐analysis, we systematically evaluated the relationship between upregulated lncRNA expression levels and clinicopathological features, five‐year survival rate, and progression‐free survival (PFS).

**Methods:**

A systematic search was performed across seven reputable databases, namely the China National Knowledge Infrastructure, Wanfang, Wipu, PubMed, Web of Science, Cochrane Library, and Embase, encompassing the period from the inception of each database until November 27, 2022. Heterogeneity among the studies was assessed through the application of Cochran's Q and *I* ^2^ statistics. All statistical analyses were conducted using Stata 14.0 software.

**Results:**

This study encompassed 30 clinical studies, involving a total of 2469 EC patients, and examined the expression of 24 lncRNAs, which were upregulated in EC samples. EC patients with higher expression of lncRNAs showed a later FIGO stage (OR = 1.94, 95% CI: 1.29 ~ 2.91), a poorer histological grade (OR = 3.40, 95% CI: 2.51 ~ 4.60), earlier deep myometrial invasion (OR = 2.57, 95% CI: 1.94 ~ 3.41), a higher likelihood of lymphatic vascular space infiltration (OR = 2.86, 95% CI: 1.15 ~ 7.14), an increased propensity for lymph node metastasis (OR = 2.89, 95% CI: 1.82 ~ 4.60), and a greater likelihood of distant metastasis (OR = 2.39, 95% CI: 1.33 ~ 4.30). All of these were statistically significant (*p* < 0.05). Furthermore, EC patients with a higher expression level of lncRNAs were significantly associated with five‐year survival (*p* < 0.05) and PFS (*p* < 0.05).

**Conclusions:**

High expression levels of upregulated lncRNAs in EC patients are associated with unfavorable clinicopathological features, a poor five‐year survival rate, and PFS. It serves as a detrimental prognostic factor and might be a biomarker and therapeutic target for EC.

## 1 Introduction

In recent years, there has been a notable rise in the prevalence of endometrial carcinoma (EC), positioning it as one of the prevailing malignancies within the female reproductive system. This surge in cases may be primarily attributed to the diminishing fertility rates and the escalating rates of obesity [1]. A majority of EC patients are typically diagnosed during the initial stages, and early intervention can significantly enhance the prognosis for these individuals. Nevertheless, a subset of patients continues to experience delayed diagnosis of their condition due to the presence of atypical clinical symptoms, leading to the proliferation of cancer cells within the local area or their dissemination to distant sites. In the context of poor prognosis for advanced‐stage or recurrent EC, there is an urgent need to develop breakthrough therapeutic strategies based on genomics. With the advent of precision oncology, the prognostic information provided by the molecular classification of EC has driven the personalization of clinical management [2]. However, biomarkers have been detected in an endless stream, and there is an increasing emphasis on the integration of molecular signatures in EC [3].

Long non‐coding RNA (lncRNA) denotes a subclass of non‐coding RNA molecules surpassing a length of 200 bp. They are characterized by the absence of an open reading coding frame and demonstrate pronounced tissue specificity [4]. LncRNAs are not only crucial in facilitating cell differentiation, apoptosis, and cycle regulation, but they also actively contribute to tumor initiation, progression, invasion, metastasis, and resistance to chemotherapeutic drugs, employing intricate mechanisms. A large number of specific up‐ or down‐regulated lncRNAs were detected in EC. H19 exhibits heightened expression in EC, thereby facilitating the proliferation, invasion, and migration of EC cells [5]. In contrast, the aberrantly reduced expression of Gas5 correlates with tumor staging and unfavorable prognosis in EC [6]. HOTAIR has been found to modulate the cisplatin resistance of EC cells through its impact on the expression of becklin‐1, multidrug resistance, and P‐glycoprotein, as well as its ability to enhance autophagy activity [7]. Therefore, it is difficult for some specific lncRNA to explain the complex molecular mechanism network during the occurrence and development of EC. We need to integrate the regulatory role of lncRNAs in EC through bioinformatics and other methods to better guide clinical diagnosis, treatment, and prognostic value.

The association between abnormal lncRNA expression and the prognostic value of EC remains a topic of debate currently. LncRNAs with different characteristics have the same or different prognostic values. A study screened out 13 immune‐related lncRNAs in the TCGA database, which can accurately predict 1‐, 2‐, 3‐, and 5‐year disease‐free survival of EC patients [8]. Based on this feature, the immune status and tumor mutation burden of EC samples can be distinguished [8]. Another study developed a genomic instability‐related lncRNA signature to evaluate the prognosis, influence immune status, and chemotherapeutic drug sensitivity in EC [9]. Six pyroptosis‐related lncRNAs also provided good prediction of EC survival, which was associated with the level of immune infiltration and T cell exhaustion [10]. It can be seen that the majority of studies in this field are characterized by a single‐center nature, small sample sizes, and limited availability of high‐quality evidence‐based medicine. In order to explore the potential of lncRNA as a prognostic biomarker for EC, we undertook a systematic evaluation through meta‐analysis to investigate the association between clinicopathological features and the expression of lncRNA, as well as its prognostic significance.

## 2 Materials and Methods

### 2.1 Search Strategy

We prospectively registered the systematic review and meta‐analysis with PROSPERO(CRD42023461550). This systematic review and meta‐analysis was conducted in accordance with PRISMA (Pre‐ferred Reporting Items for Systematic Reviews and Meta‐Analyses) guidelines [11].

The papers were obtained from widely used literature databases, such as China National Knowledge Infrastructure (CNKI), Wanfang, Wipu, PubMed, Web of Science, Cochrane Library, and Embase, until November 27, 2022. The following search terms were used: “Endometrial Neoplasms or Endometrial Neoplasm or Neoplasm, Endometrial or Neoplasms, Endometrial or Endometrial Carcinoma or Carcinoma, Endometrial or Carcinomas, Endometrial or Endometrial Carcinomas or Endometrial Cancer or Cancer, Endometrial or Cancers, Endometrial or Endometrial Cancers or Endometrium Cancer or Cancer, Endometrium or Cancers, Endometrium or Cancer of the Endometrium or Carcinoma of Endometrium or Endometrium Carcinoma or Endometrium Carcinomas or Cancer of Endometrium or Endometrium Cancers” and “RNA, Long Noncoding or Noncoding RNA, Long or lncRNA or Long ncRNA or ncRNA, Long or RNA, Long Non‐Translated or Long Non‐Translated RNA or Non‐Translated RNA, Long or RNA, Long Non Translated or Long Non‐Coding RNA or Long Non Coding RNA or Non‐Coding RNA, Long or RNA, Long Non‐Coding or Long Non‐Protein‐Coding RNA or Long Non Protein Coding RNA or Non‐Protein‐Coding RNA, Long or RNA, Long Non‐Protein‐Coding or Long Noncoding RNA or RNA, Long Untranslated or Long Untranslated RNA or Untranslated RNA, Long or Long ncRNAs or ncRNAs, Long or Long Intergenic Non‐Protein Coding RNA or Long Intergenic Non Protein Coding RNA or LincRNA or LINC RNA or LincRNAs”. To ensure comprehensive coverage, relevant references in the literature were manually searched to minimize the risk of overlooking any relevant studies. The document type was restricted to English and Chinese.

### 2.2 Selection Criteria

The inclusion criteria were as follows: (1)This study aims to investigate the association between elevated expression of lncRNAs and clinicopathological characteristics as well as the prognosis of EC through a cohort design; (2) Patients diagnosed with EC were excluded if they had received preoperative radiotherapy, chemotherapy, or endocrine therapy; (3) The participants were categorized into high and low expression groups based on the levels of lncRNAs; (4) Outcome indicators encompass various clinical features such as age, FIGO stage, histological grade, myometrial invasion, lymphatic vascular space invasion, lymph node metastasis, and distant metastasis. Additionally, prognosis is evaluated through parameters such as the five‐year survival rate, progression‐free survival (PFS), hazard risk ratio (HR) or odds ratio (OR), and their corresponding 95% confidence interval (CI); (5) The literature included in this study comprises publications in both Chinese and English languages.

The exclusion criteria were as follows: (1) A study involving experimentation on cells or animals; (2) Correspondence in the form of letters, case reports, reviews, meeting minutes, and comments; (3) The necessary data could not be extracted from the original article due to the unavailability of the full text or duplication of publication; (4) A study that reported the presence of multiple lncRNAs; (5) Investigation focused on reprocessing data obtained from publicly accessible databases, such as the GEO database and TCGA database; (6) In the case of multiple publications reporting the same subset of data, only the most recent publications are considered.

### 2.3 Data Extraction and Quality Assessment

The process of data extraction was conducted by two researchers in an independent manner, with any disagreements being resolved by a third researcher. The extracted information encompasses various aspects, including the first author, year of publication, country of origin, specimen type, type of lncRNAs, method of detection, sample size (categorized as high or low), cutoff value for lncRNAs expression, indices related to outcomes (such as age, FIGO stage, histological grade, myometrial invasion, lymphatic vascular space invasion, lymph node metastasis, distant metastasis, five‐year survival rate, and PFS), follow‐up duration, and survival analysis. The HR and its corresponding 95% CI of the five‐year survival rate or PFS period can be obtained by directly extracting the relevant data from the survival data presented in this paper. Alternatively, they can be indirectly calculated from the survival curve using Engauge Digitizer software. The quality of the literature included in the study was assessed using the Newcastle‐Ottawa scale (NOS). A NOS score of ≥ 6 indicates a high‐quality study.

### 2.4 Statistical Analysis

The Stata14.0 software was utilized to conduct a quantitative synthesis and comprehensive analysis of Meta. The clinicopathological features were examined using the OR value and corresponding 95% CI, while the impact of lncRNA on the prognosis of patients with EC was assessed by combining HR and 95% CI. The evaluation criteria consisted of the five‐year survival rate and PFS. The heterogeneity of the included studies was evaluated through the utilization of the Q test and I^2^ test. When the heterogeneity of each study is small (*I* ^2^ < 50%; *p* > 0.05), the fixed effect model is employed for Meta‐analysis. Conversely, if there is substantial heterogeneity (*I* ^2^ > 50%; *p* ≤ 0.05), the random effect model is utilized for meta‐analysis. In the event of significant heterogeneity, sensitivity analysis is employed to assess the stability of the merged results. Additionally, subgroup analysis can be employed to investigate the impact of heterogeneity sources and grouping factors on the outcomes. The funnel plot and Egger test were employed to assess publication bias.

## 3 Results

### 3.1 Study Selection

**FIGURE 1:**
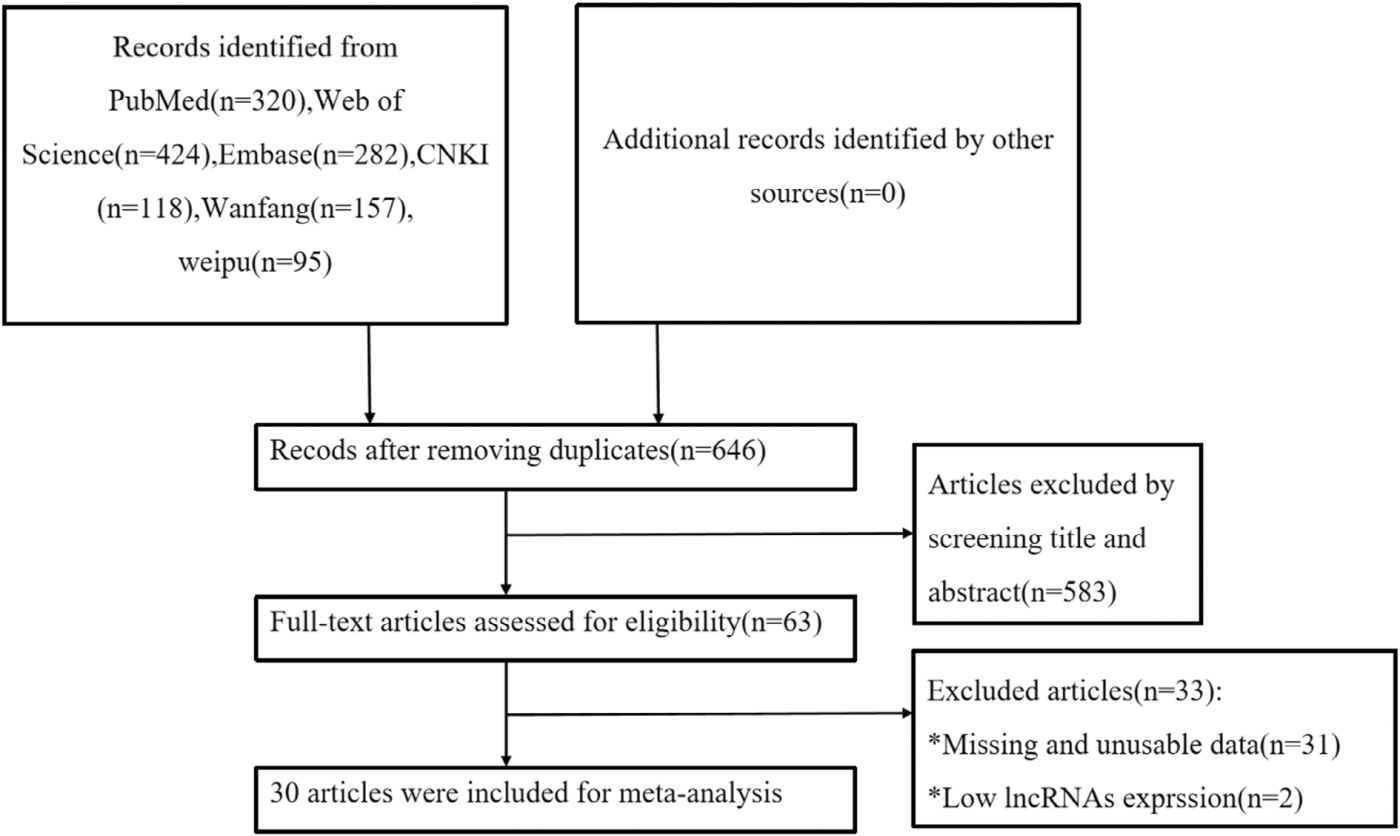
The flowchart of the selection.

As shown in Figure 1, a total of 1400 papers were obtained from 7 databases. Out of these, 754 papers were eliminated due to duplication, employing both automated and manual methods. Additionally, 583 papers were excluded based on a preliminary assessment of their titles or abstracts. The complete texts of the remaining 63 papers were meticulously examined, resulting in the exclusion of 33 studies due to insufficient expression of lncRNAs or inadequate data. Ultimately, a final selection of 30 clinical cohort studies was included in the systematic review and meta‐analysis (Figure 1) [6, 12, 13, 14, 15, 16, 17, 18, 19, 20, 21, 22, 23, 24, 25, 26, 27, 28, 29, 30, 31, 32, 33, 34, 35, 36, 37, 38, 39, 40].

### 3.2 Characteristics of Included Studies

We conducted a comprehensive review of 30 studies [6, 12, 13, 14, 15, 16, 17, 18, 19, 20, 21, 22, 23, 24, 25, 26, 27, 28, 29, 30, 31, 32, 33, 34, 35, 36, 37, 38, 39, 40] presented in Table 1, comprising 13 Chinese papers and 17 English papers. These studies encompassed 24 distinct lncRNAs, namely ZNFX1‐AS1, ZFASI, UCA1, SNHG1, PCAT1, NNT‐AS1, NEAT1, MCM3AP‐AS1, MALAT1, LINPI, LINC00958, LINC00565, LINC00393, HOTAIR, H19, DLEU1, DCST1‐AS1, CASC9, CARLo‐5, BANCR, AFAP1‐AS1, AFAP1‐AS, LINC01123, and HOXB‐AS1. The collective sample size across these studies amounted to 2469 patients diagnosed with EC, spanning the period from 2013 to 2022. With the exception of one study, the samples originated from Poland, while the remaining studies were conducted in China. One sample was derived from serum, while the remaining samples were obtained from tissue. There is no universally accepted criterion for determining the diagnostic expression level cutoff currently. These lncRNAs, which showed an up‐regulated trend in EC, were divided into high and low expression groups. A total of 21 studies furnished data on clinicopathological features, while 17 studies examined the correlation between lncRNA and five‐year survival. Additionally, 2 studies evaluated the association between lncRNA and PFS. All studies obtained a minimum Newcastle‐Ottawa Scale (NOS) score of six, as indicated in Table 2.

**TABLE 1:** Characteristics of studies included in this meta‐analysis.

Author (particular year)	Country	Specimen type	LncRNAs typology	Inspection methods	Sample size (max/min)	Truncation value	Outcome indicator	Outcome indicator	Follow‐up time (months)
He (2013) [6]	China	Organization	HOTAIR	ISH	63/24	NA	①②③④⑤⑥	NA	NA
Huang (2014) [12]	China	Organization	HOTAIR	qRT‐PCR	61/20	2.6	①②③④⑤⑥	NA	NA
Han (2016) [14]	China	Organization	MALAT1	qRT‐PCR	104	Average value	⑨	Univariate analysis	48
Zhao XW (2016) [15]	China	Organization	CARLo‐5	qRT‐PCR	54/54	Upper quartile	①②③④⑤⑥	NA	NA
Lu (2016) [16]	China	Organization	UCA1	qRT‐PCR	12/33	Upper quartile	①②⑥⑦⑧	Univariate analysis	60
Wang D (2016) [17]	China	Organization	BANCR	qRT‐PCR	15/15	Upper quartile	②④⑥	NA	NA
Łuczak (2016) [18]	Poland	Organization	HOTAIR	qRT‐PCR	56/100	Upper quartile	②	NA	NA
Shen (2017) [19]	China	Organization	NEAT1	ISH	19/39	Upper quartile	①②③⑥⑦⑧	Univariate analysis	60
Guan (2019) [20]	China	Organization	CARLo⁃5	qRT‐PCR	79	NA	⑧	Multivariate statistics	60
Yang L (2019) [21]	China	Organization	AFAP1‐AS1	qRT‐PCR	64/56	Average value	③④⑥⑧	Multivariate statistics	60
Wu (2019) [22]	China	Organization	H19	qRT‐PCR	56/34	NA	①②③④⑥	NA	NA
Mai (2019) [23]	China	Organization	LINC00393	qRT‐PCR	117/33	NA	②⑥	NA	NA
Zhao XH (2019) [25]	China	Organization	PCAT1	qRT‐PCR	50/39	Upper quartile	①②④⑤⑥⑧	Multivariate statistics	60
Wang F (2020) [24]	China	Organization	ZFASI	qRT‐PCR	45/31	1.72	⑧	Multivariate statistics	60
Wang S (2020) [26]	China	Organization	AFAP1‐AS	qRT‐PCR	62/50	Average value	②③④⑥⑧	Multivariate statistics	
Shan (2020) [27]	China	Plasma	DLEU1	qRT‐PCR	128	Upper quartile	⑧	Multivariate statistics	60
Yang YG (2020) [28]	China	Organization	LINC01123	qRT‐PCR	52/54	Upper quartile	②③④⑥⑧	Multivariate statistics	60
Sun (2021) [29]	China	Organization	ZNFX1‐AS1	qRT‐PCR	41/23	Upper quartile	①②④⑥⑧	Multivariate statistics	60
Yin (2020) [30]	China	Organization	LINC00565	qRT‐PCR	26/26	Upper quartile	⑥⑦	NA	NA
Ke (2020) [31]	China	Organization	DCST1‐AS1	qRT‐PCR	31/31	Upper quartile	⑧	Univariate analysis	60
Wang GF (2020) [32]	China	Organization	SNHG1	qRT‐PCR	26/26	NA	⑥⑦	NA	NA
Guo (2021) [39]	China	Organization	LINPI	qRT‐PCR	41/41	Upper quartile	③⑤⑥	NA	NA
Li (2021) [33]	China	Organization	LINC00958	qRT‐PCR	50/50	NA	⑧	Univariate analysis	60
Yu (2021) [34]	China	Organization	MCM3AP‐AS1	qRT‐PCR	30/30	Upper quartile	⑥⑧⑨	Univariate analysis	60
Ma (2021) [36]	China	Organization	NNT‐AS1	qRT‐PCR	24/34	1.7	⑧	Multivariate statistics	60
Wang TT (2021) [37]	China	Organization	CASC9	qRT‐PCR	46/34	1.42	⑧	Multivariate statistics	60
Gong (2022) [35]	China	Organization	AFAP1‐AS	qRT‐PCR	62/50	Average value	②③④⑥⑧	Multivariate statistics	60
Liu D (2020) [40]	China	Organization	HOXB‐AS1	qRT‐PCR	32/32	NA	②⑥	NA	NA
Luo (2022) [13]	China	Organization	NEAT1	qRT‐PCR	18/18	Upper quartile	①②③④⑤⑥	NA	NA
Liu YJ (2022) [38]	China	Organization	SNHG1	qRT‐PCR	27/27	1.61	⑧	Univariate analysis	60

*Note:* ① Age; ② FIGO stage; ③ Histological grade; ④ Myometrial invasion; ⑤ Lymphovascular space invasion; ⑥ Lymph node metastasis; ⑦ Distant metastasis; ⑧ Five‐year survival rate; ⑨ progression‐free survival.

Abbreviations: ISH, in situ hybridization; qRT‐PCR: quantitative reverse transcription‐polymerase chain reaction.

**TABLE 2:** Quality assessment of the individual study.

Study cohort	Cohort selection criteria				Comparability	Study assessment			Score
	Representativeness of exposed cohort	Selection of nonexposed cohort	Ascertainment of exposure	Outcome not present at the start		Assessment of outcome	Follow‐up length	Follow‐up adequacy	
Gong (2022) [35]	*	*	*	*	**	*	*	—	8
Guan (2019) [20]	*	*	*	*	*	*	*	*	8
Guo (2021) [39]	*	*	*	*	**	*	—	*	8
Han (2016) [14]	*	*	*	*	*	*	—	—	6
Luo (2022) [13]	*	*	*	*	*	*	*	*	8
Ma (2021) [36]	*	*	*	*	**	*	*	*	9
Mai (2019) [23]	*	*	*	*	*	*	—	—	6
Wang S (2020) [26]	*	*	*	*	**	*	*	—	8
Wu (2019) [22]	*	*	*	*	**	*	*	—	8
Liu YJ (2022) [38]	*	*	*	*	**	*	*	*	9
Wang F (2020) [24]	*	*	*	*	**	*	*	*	9
Wang TT (2021) [37]	*	*	*	*	**	*	*	*	9
Yang L (2019) [21]	*	*	*	*	**	*	*	*	9
Lu (2016) [16]	*	*	*	*	—	*	*	*	7
Shan (2020) [27]	*	*	*	*	**	*	*	—	8
Shen (2017) [19]	*	*	*	*	—	*	*	*	7
Wang D (2016) [17]	*	*	*	*	**	*	—	—	7
Yin (2020) [30]	*	*	*	*	*	*	*	—	7
Zhao XH (2019) [25]	*	*	*	*	**	*	*	—	8
Zhao XW (2016) [15]	*	*	*	*	*	*	*	—	7
Li (2021) [33]	*	*	*	*	*	*	*	*	8
Yu (2021) [34]	*	*	*	*	*	*	*	*	8
He (2013) [6]	*	*	*	*	*	*	—	—	6
Huang (2014) [12]	*	*	*	*	*	*	—	—	6
Ke (2020) [31]	*	*	*	*	*	*	*	*	8
Liu D (2020) [40]	*	*	*	*	*	*	—	—	6
Łuczak (2016) [18]	*	*	*	*	*	*	*	—	7
Sun (2021) [29]	*	*	*	*	*	*	*	*	8
Wang GF (2020) [32]	*	*	*	*	*	*	*	—	7
Yang YG (2020) [28]	*	*	*	*	*	*	*	—	7

*Note:* For comparability, two asterisks are obtained if the multivariate analysis is used to correct age‐mixed factors. If the age is not fixed and there is no significant difference in age, ISS stage, etc., an asterisk is given; otherwise, 0 asterisks are obtained. For follow‐up adequacy, if the loss rate to follow‐up is < 20%, an asterisk is scored. If the rate of loss to follow‐up is not described or ≥ 20%, 0 asterisks are obtained.

### 3.3 Correlation of lncRNA Expression With Clinicopathological Characteristics of EC

**FIGURE 2:**
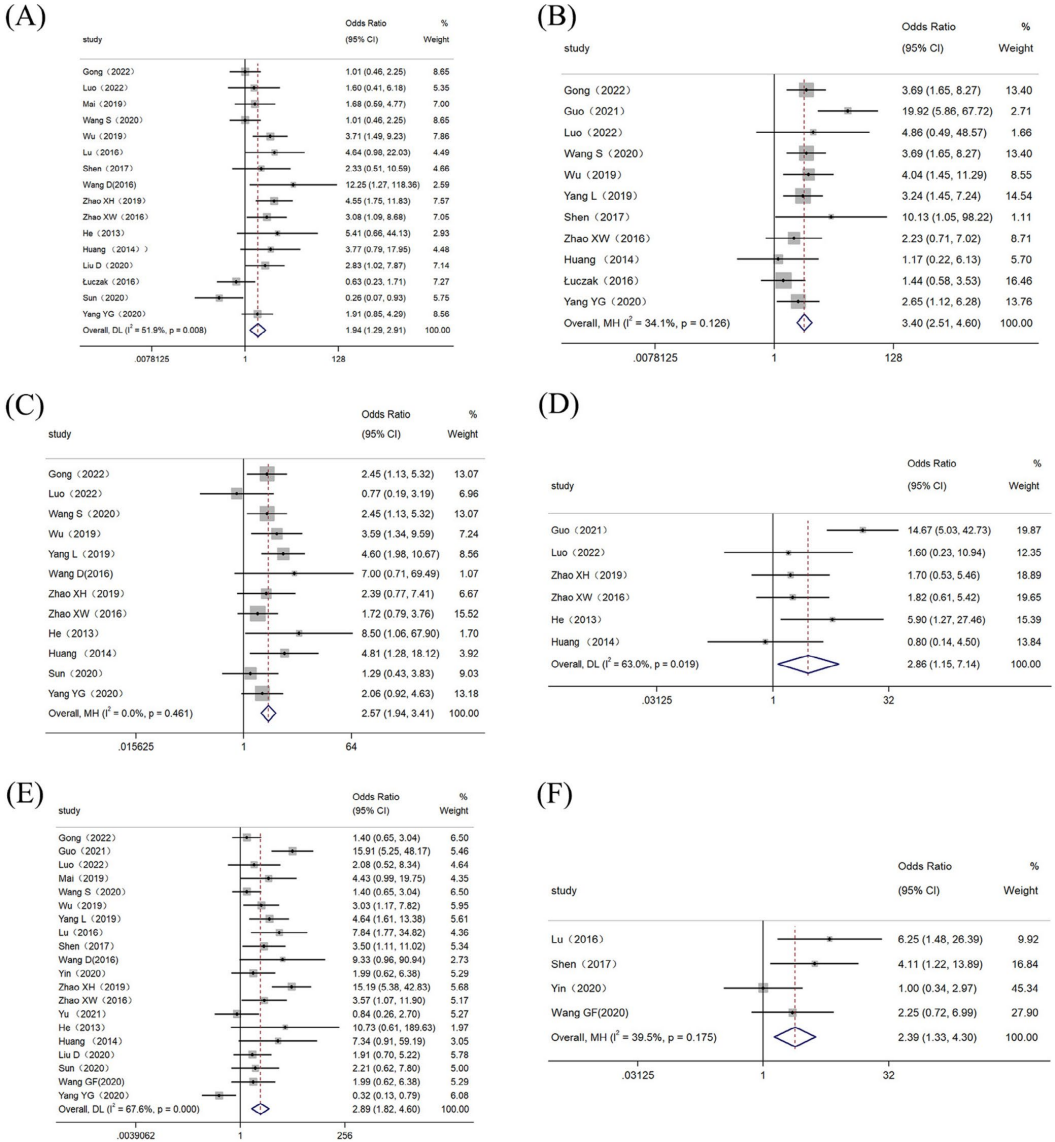
Forest plot for the association between the high expression of lncRNAs and clinical features. (A) FIGO stage. (B) Histological grade. (C) Myometrial invasion. (D) Lymphovascular space invasion. (E) Lymph node metastasis. (F) Distant metastasis.

**FIGURE 3:**
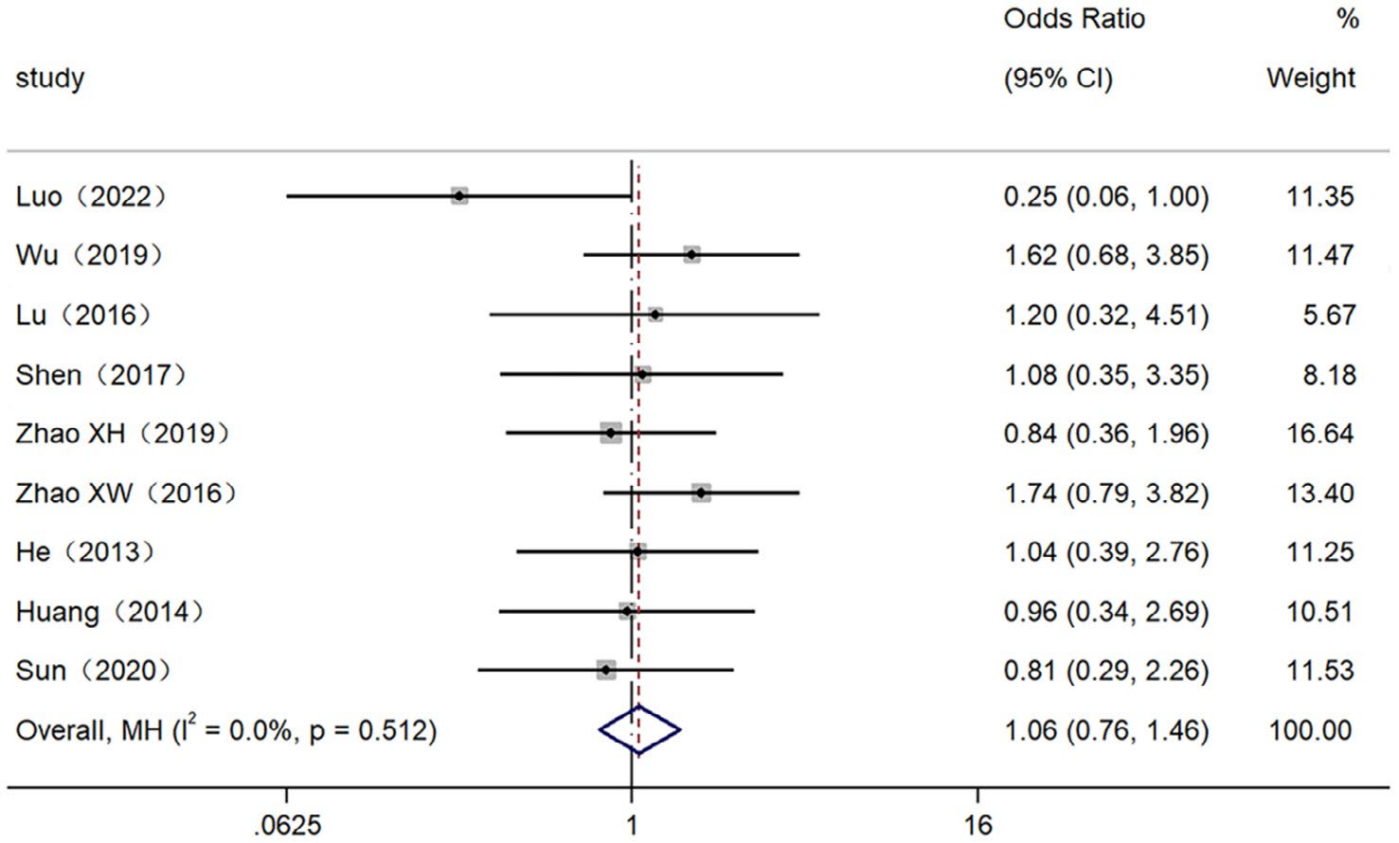
Forest plot of the high expression of LncRNAs with the clinical characteristic of age.

There were 21 relevant studies included in this meta‐analysis with clinicopathological features. This analysis included data from 1754 patients, and the appropriate effect model was selected based on *I* ^2^ and *p* values (Table 3). EC patients with higher expression of lncRNAs showed a later FIGO stage (OR = 1.94, 95% CI: 1.29 ~ 2.91) (Figure 2A), a poorer histological grade (OR = 3.40, 95% CI: 2.51 ~ 4.60) (Figure 2B), earlier deep myometrial invasion (OR = 2.57, 95% CI: 1.94 ~ 3.41) (Figure 2C), a higher likelihood of lymphatic vascular space infiltration (OR = 2.86, 95% CI: 1.15 ~ 7.14) (Figure 2D), an increased propensity for lymph node metastasis (OR = 2.89, 95% CI: 1.82 ~ 4.60) (Figure 2E), and a greater likelihood of distant metastasis (OR = 2.39, 95% CI: 1.33 ~ 4.30) (Figure 2F). The observed differences were found to be statistically significant (*p* < 0.05). There was no significant difference in age between the high and low lncRNA expression groups (*p* = 0.746 > 0.05) (Figure 3).

**TABLE 3:** Summary of high lncRNAs expressed related to clinicopathological features of EC.

Clinicopathological feature	No. of studies	No. of patients	Meta analysis		Heterogeneity		Effect model
			OR (95% CI)	*p*	*I* ^2^ (%)	*p*	
Age (≥ 55/< 55)	9	652	1.06 (0.76, 1.46)	0.746	0.00%	0.512	Fixed
FIGO stage (III + IV/I + II)	16	1385	1.94 (1.29, 2.91)	0.002	51.90%	0.008	Random
Histological grade (G3/G1 + G2)	11	1057	3.40 (2.51, 4.60)	0	34.10%	0.126	Fixed
Myometrial invasion (≥ 1/2/< 1/2)	12	1035	2.57 (1.94, 3.41)	0	0.00%	0.461	Fixed
Lymphovascular space invasion (YES/NO)	6	483	2.86 (1.15, 7.14)	0.024	63.00%	0.019	Random
Lymph node metastasis (YES/NO)	20	1598	2.89 (1.82, 4.60)	0	67.60%	0	Random
Distant metastasis (YES/NO)	4	207	2.39 (1.33, 4.30)	0.004	39.50%	0.175	Fixed

### 3.4 Heterogeneity Analysis and Subgroup Analysis

**FIGURE 4:**
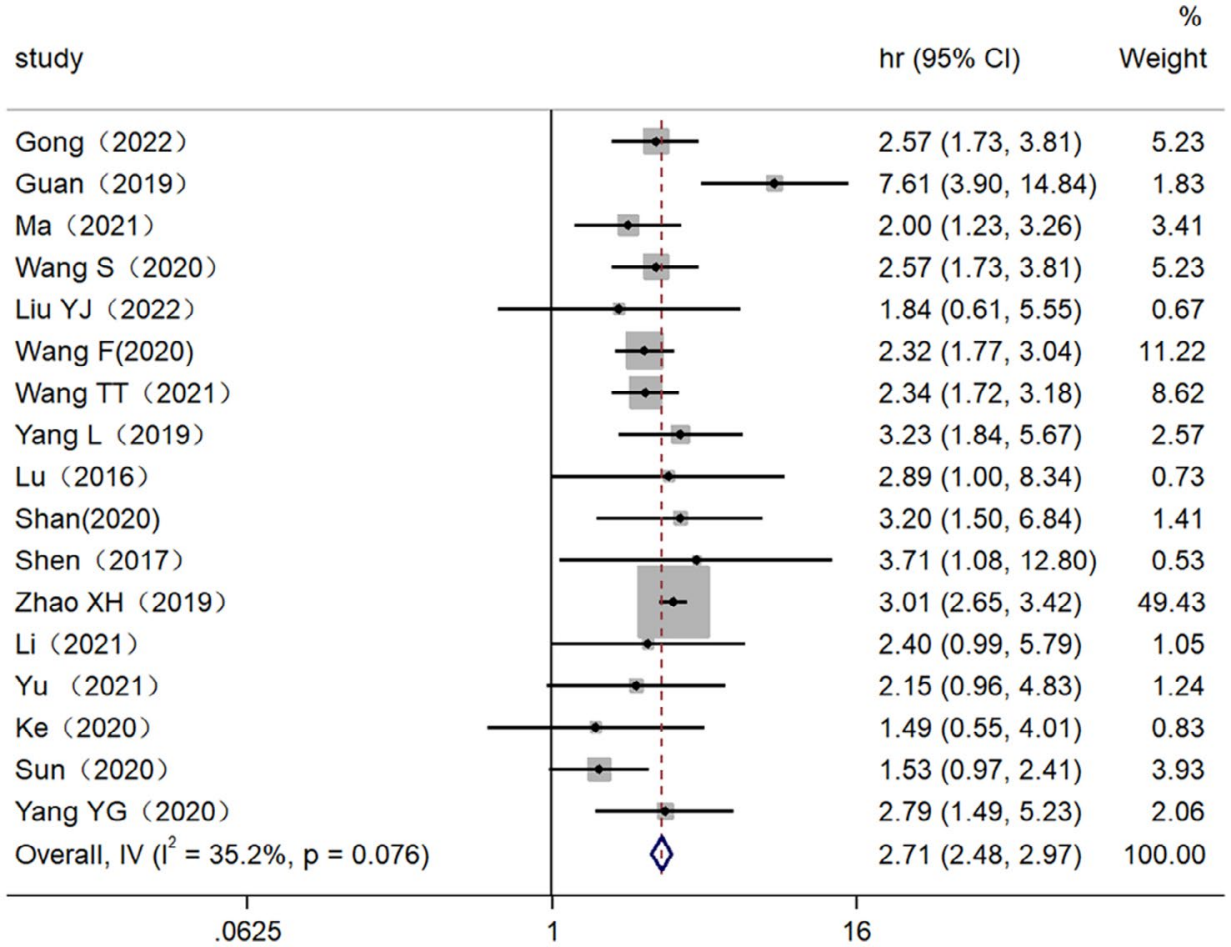
Forest plot for the association between high lncRNAs expression and five‐year survival rate.

In order to explore the relationship between high lncRNA expression and 5‐year survival, 17 original studies involving 1403 patients diagnosed with EC were analyzed. The heterogeneity analysis revealed a low level of heterogeneity among the studies (*I* ^2^, 35.2%, *p* = 0.076 > 0.05), leading to the adoption of a fixed effect model. There was a significant correlation between high expression levels of lncRNAs and the five‐year survival rate (HR = 2.71, 95% CI: 2.48–2.97) (*p* = 0.000 < 0.05) (Figure 4). Based on various survival analysis methods (univariate analysis or multivariate analysis), the examination of sample size (sample size ≥ 100 or < 100), and the determination of the cutoff value for lncRNA expression level (median, mean or NA), the findings indicate a significant disparity between high expression of lncRNAs and the five‐year survival rate. The potential sources of heterogeneity include multi‐factor analysis (*I* ^2^ = 54.8%, *p* = 0.015), sample size < 100 (*I* ^2^ = 58.2%, *p* = 0.08), and NA (*I* ^2^ = 59.1%, *p* = 0.032) in relation to the cutoff value (Table 4).

**TABLE 4:** Subgroup analysis of high lncRNA expression and five‐year survival rate in EC.

Subgroup	Number of studies (*n*)	Number of sample (*n*)	Meta analyze heterogeneity			
			HR (95% CI)	*p*	*I* ^2^	*p*
Survival analysis						
Multivariate	11	1024	2.63 (2.21, 3.13)	0.000	54.8%	0.015
Univariate	6	379	2.24 (1.50, 3.35)	0.000	0.0%	0.893
Sample size						
≥ 100	6	678	2.72 (2.19, 3.37)	0.000	0.0%	0.979
< 100	11	725	2.71 (2.45, 2.99)	0.000	58.2%	0.008
Truncation value						
Upper quartile	8	612	2.50 (1.95, 3.21)	0.000	31.6%	0.176
Average value	3	344	2.69 (2.09, 3.45)	0.000	0.0%	0.775
NA	6	447	2.62 (1.90, 3.62)	0.000	59.1%	0.032

Abbreviation: NA, not mentioned.

**FIGURE 5:**
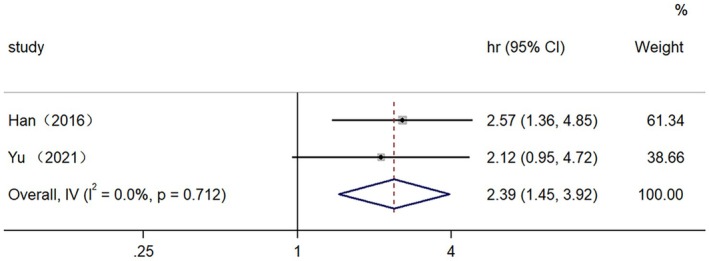
Sensitivity analysis of five‐year survival rate.

The relationship between high expression of lncRNAs and PFS in patients with EC was investigated in two studies. Heterogeneity analysis revealed no significant heterogeneity among the studies (*I* ^2^ = 0.0%, *p* = 0.712 > 0.05), the fixed effect model was employed. The findings from the Meta‐analysis indicate that patients with high expression of lncRNAs had a significantly shorter PFS compared to those with low expression (HR = 2.39, 95% CI: 1.45–3.92) (Figure 5). This difference was statistically significant, as evidenced by (*z* = 3.428, *p* = 0.001).

### 3.5 Sensitivity Analysis

**FIGURE 6:**
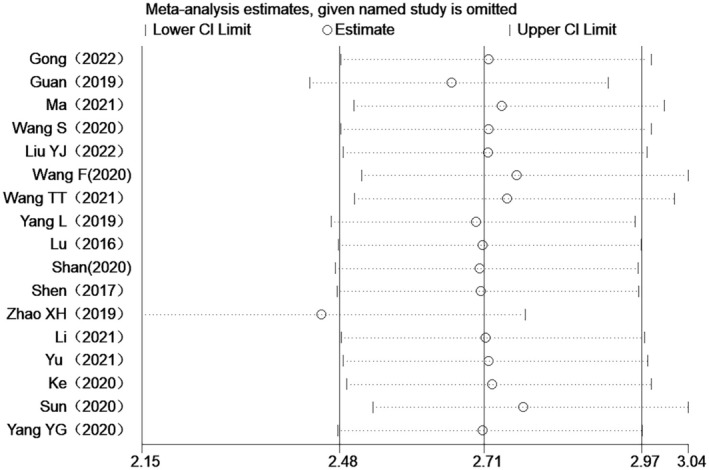
Forest plot for the association between high lncRNAs expression and PFS.

In order to assess the influence of individual studies on the overall hazard ratio (HR), a sensitivity analysis was conducted by excluding each study from the aggregate analysis. The results indicate that the overall HR remains stable, suggesting that the meta‐analysis results are robust (Figure 6).

### 3.6 Publication Bias

**FIGURE 7:**
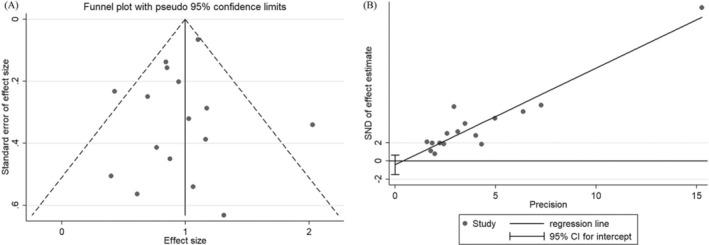
Publication bias. (A) Funnel plot analysis. (B) Begg's funnel plot.

The published offset assessment was conducted by employing the funnel chart to qualitatively assess the publication offset of the Meta‐analysis. The analysis of the funnel chart distribution of the five‐year survival rate exhibited symmetrical patterns on both the left and right sides (Figure 7A). In addition, the Egger test showed that there was no substantial evidence to support the existence of publication bias (*p* > 0.420) (Figure 7B).

## 4 Discussion

Due to the unfavorable prognosis linked to advanced and metastatic EC, it has become crucial to explore tumor markers that can facilitate early detection and intervention. Extensive investigation has revealed the important role of lncRNAs in regulating EC development, progression and prognosis [5, 6, 7, 22, 25, 41]. However, there is no consensus on the prognostic significance of aberrant expression of different types of lncRNAs in EC. Moreover, the regulatory mechanism of a large number of lncRNAs involved in the occurrence, development, and prognosis of EC cannot be explained by one of them. With the advancements in high‐throughput sequencing technology and the emergence of targeted therapy in recent years, research on aberrant lncRNA expression has become more diverse and precise. In the past 5 years, no studies have explored the relationship between elevated lncRNA expression and clinicopathological features and prognosis in EC patients. A previous study focused only on overall survival time and did not explore the association between high lncRNA expression and other survival outcomes [42]. Therefore, in order to conduct a wider range of investigation, our meta‐analysis incorporates a substantial quantity of high‐quality studies and employs a broader range of outcome indicators to enhance the dependability of the findings.

Age, FIGO stage, myometrial invasion, histological grade, lymphatic vascular space invasion, lymph node metastasis, and distant metastasis serve as crucial benchmarks for assessing patient prognosis. So we incorporated these projects as discernible indicators of clinicopathologic characteristics in order to evaluate the association between elevated expression of lncRNAs and the prognosis of EC. The results showed that worse clinicopathologic features in EC patients, except for age, were associated with higher levels of upregulated lncRNAs expression. In addition, we used five‐year survival and PFS as prognostic survival indicators compared to previous studies. In EC patients, upregulated lncRNAs expression was higher, with lower five‐year survival rates and PFS. Consequently, elevated expression levels of upregulated lncRNAs may serve as an indicator of survival rate and poor overall prognosis in EC patients.

The results imply that for EC patients with upregulated lncRNAs expression, molecular targeted inhibition of lncRNAs can be achieved to reduce the pathological grade, alleviate drug resistance, and prolong the prognosis. The lncRNAs, like H19, NEAT1, and ZEB1‐AS1, included in our study have been shown to be involved in various signaling pathways related to EC. The increased expression of H19 has been shown to enhance the mobility and invasiveness of EC cells by modulating metastatic genes (Hmga2, c‐Myc, and Imp3) through let‐7‐mediated mechanisms [43]. In recent years, there has been increasing interest in the role of exosome‐associated lncRNAs in cancer diagnosis and treatment. The exocrine NEAT1, originating from cancer‐associated fibroblasts, can be transferred to EC cells. Then, NEAT1 played a role in promoting the carcinogenicity of EC by modulating the miR‐26a/b‐5p‐STAT3‐YKL‐40 axis [44]. In terms of EC cells chemodrug resistance, one study showed that the upregulation of ZEB1‐AS1 promoted the proliferation and lymph node metastasis of EC cells while reducing sensitivity to chemotherapeutic agents such as 5‐fluorouracil and cisplatin [45].

The above articles reflect a strong correlation between high expression levels of upregulated lncRNA and the development, progression, and poor prognosis of EC, which is consistent with the conclusions of this study. At the same time, it also enlightens us to think about how to apply the controllability of lncRNAs to the clinical diagnosis and treatment of EC patients. Due to the predictability of the lncRNA model on the immune microenvironment of EC patients, it holds great promise in immunotherapy [8, 9, 10]. In addition, lncRNAs can achieve signal transduction between tumor cells through exosomes, and engineering exosomes carrying lncRNAs can achieve precise regulation that reduces cytotoxicity and significantly inhibits tumor growth [46, 47]. It really helps us develop drugs that specifically target these lncRNAs and inhibit their role in tumor progression. On the other hand, we can incorporate the identified lncRNAs associated with poor prognosis into diagnostic protocols for endometrial cancer, allowing clinicians to better stratify patients according to their risk profile and thus develop more precise and personalized treatment strategies. Additionally, we can include patients with high expression of these lncRNAs in a more rigorous follow‐up program, improving the efficiency of early detection and intervention in the disease and thus improving overall survival.

Although our meta‐analysis included as many high‐quality studies as possible, there were some limitations. Since the majority of participants were of Chinese descent and the samples relied on pathological tissue specimens, this limited the generality of the findings and conclusions to a certain extent. Furthermore, some of the studies included in this analysis did not explicitly provide the necessary prognostic indicator data, so the above information needed to be manually extracted from the Kaplan–Meier survival curve. In the future, there is a need to expand the sample size as much as possible and update the content by supplementing multi‐region and multi‐species lncRNA studies.

In summary, our study synthesized multiple small samples into a large sample for statistical analysis, first showing that the high expression of lncRNAs is significantly associated with poor clinicopathological characteristics and unfavorable prognosis (five‐year survival rate and PFS) in EC patients. The reliability of lncRNAs as potential prognostic biomarkers is greatly enhanced. Our study provides evidence to support the future application of lncRNAs in the diagnosis, monitoring, and follow‐up strategies of EC patients, as well as the development of targeted therapies.

## Author Contributions

**Xiaotong Zhao:** conceptualization (lead), data curation (lead), formal analysis (lead), writing – original draft (equal). **Ziling Yang:** methodology (lead), writing – original draft (equal), writing – review and editing (lead). **Tianjiao Zheng:** data curation (lead), formal analysis (equal), writing – original draft (equal). **Mengyao Zeng:** validation (lead). **Xiaowen Lin:** funding acquisition (equal). **Huixin Chen:** funding acquisition (equal). **Weiqin Zheng:** funding acquisition (equal). **Sizheng Peng:** conceptualization (supporting). **Shibo Li:** conceptualization (supporting). **Tao Song:** resources (equal), supervision (equal). **Yuhui Sun:** resources (equal), supervision (equal).

## Ethics Statement

This article does not contain any studies with human participants or animals performed by any author.

## Conflicts of Interest

The authors declare no conflicts of interest.

## Data Availability

The data that support the findings of this study are available from the corresponding author upon reasonable request.

## References

[1] R. L. Siegel , K. D. Miller , N. S. Wagle , and A. Jemal , “Cancer Statistics, 2023,” CA: A Cancer Journal for Clinicians 73 (2023): 17–48, 10.3322/caac.21763. 36633525

[2] A. Jamieson , L. M. Barroilhet , and J. N. McAlpine , “Molecular Classification in Endometrial Cancer: Opportunities for Precision Oncology in a Changing Landscape,” Cancer 128 (2022): 2853–2857, 10.1002/cncr.34328. 35657171

[3] A. Jamieson and J. N. McAlpine , “Molecular Profiling of Endometrial Cancer From TCGA to Clinical Practice,” Journal of the National Comprehensive Cancer Network 21, no. 2 (2023): 210–216, 10.6004/jnccn.2022.7096. 36791751

[4] F. Kopp , “Long Noncoding RNA H19,” Journal of Gene Medicine 21 (2019): e3104, 10.1002/jgm.3104. 31177599

[5] H. Zhu , Y.‐M. Jin , X.‐M. Lyu , L.‐M. Fan , and F. Wu , “Long Noncoding RNA H19 Regulates HIF‐1α/AXL Signaling Through Inhibiting miR‐20b‐5p in Endometrial Cancer,” Cell Cycle 18 (2019): 2454–2464, 10.1080/15384101.2019.1648958. 31411527 PMC6739051

[6] X. He , W. Bao , X. Li , et al., “The Long Non‐Coding RNA HOTAIR Is Upregulated in Endometrial Carcinoma and Correlates With Poor Prognosis,” International Journal of Molecular Medicine 33 (2014): 325–332, 10.3892/ijmm.2013.1570. 24285342

[7] Z. Li , Z. Yu , X. Meng , et al., “Long Noncoding RNA GAS5 Impairs the Proliferation and Invasion of Endometrial Carcinoma Induced by High Glucose via Targeting miR‐222‐3p/p27,” American Journal of Translational Research 11 (2019): 2413–2421. 31105847 PMC6511791

[8] J. Liu , J. Mei , Y. Wang , et al., “Development of a Novel Immune‐Related lncRNA Signature as a Prognostic Classifier for Endometrial Carcinoma,” International Journal of Biological Sciences 17 (2021): 448–459, 10.7150/ijbs.51207. 33613104 PMC7893582

[9] J. Liu , G. Cui , J. Ye , Y. Wang , C. Wang , and J. Bai , “Comprehensive Analysis of the Prognostic Signature of Mutation‐Derived Genome Instability‐Related lncRNAs for Patients With Endometrial Cancer,” Frontiers in Cell and Development Biology 10 (2022): 753957, 10.3389/fcell.2022.753957. PMC9012522 35433686

[10] J. Liu , R. Geng , S. Ni , et al., “Pyroptosis‐Related lncRNAs Are Potential Biomarkers for Predicting Prognoses and Immune Responses in Patients With UCEC,” Molecular Therapy ‐ Nucleic Acids 27 (2022): 1036–1055, 10.1016/j.omtn.2022.01.018. 35228898 PMC8844853

[11] M. J. Page , J. E. McKenzie , P. M. Bossuyt , et al., “The PRISMA 2020 Statement: An Updated Guideline for Reporting Systematic Reviews,” BMJ 372 (2021): n71, 10.1136/bmj.n71. 33782057 PMC8005924

[12] J. Huang , P. Ke , L. Guo , et al., “Lentivirus‐Mediated RNA Interference Targeting the Long Noncoding RNA HOTAIR Inhibits Proliferation and Invasion of Endometrial Carcinoma Cells In Vitro and In Vivo,” International Journal of Gynecological Cancer 24 (2014): 635–642, 10.1097/IGC.0000000000000121. 24758900

[13] Y. Luo , J. T. Fan , and J. H. Yang , “Clinicopathological Significance of LncRNA‐NEAT1 in Endometrial Carcinoma and Its Value in Prognosis,” Chinese Journal of Family Planning & GYNECOTOKOLOGY 14 (2022): 71–75.

[14] C. X. Han , “The Expression and Clinical Significance of Long Non‐Coding RNA MALAT1 in Endometrial Cancer,” Chinese Journal of Family Planning & GYNECOTOKOLOGY 8, no. 6 (2016): 51–55.

[15] X. Zhao , X. Wei , L. Zhao , et al., “Silencing of UCA1,” Environmental and Molecular Mutagenesis 57 (2016): 508–515, 10.1002/em.22031. 27432114

[16] L. Lu , Y. Shen , K.‐F. Tseng , W. Liu , H. Duan , and W. Meng , “Silencing of UCA1, a Poor Prognostic Factor, Inhibited the Migration of Endometrial Cancer Cell,” Cancer Biomarkers 17 (2016): 171–177, 10.3233/CBM-160628. 27540975 PMC13020495

[17] D. Wang , D. Wang , N. Wang , Z. Long , and X. Ren , “Long Non‐Coding RNA BANCR Promotes Endometrial Cancer Cell Proliferation and Invasion by Regulating MMP2 and MMP1 via ERK/MAPK Signaling Pathway,” Cellular Physiology and Biochemistry 40 (2016): 644–656, 10.1159/000452577. 27898420

[18] A. Łuczak , A. Supernat , S. Łapińska‐Szumczyk , et al., “HOTAIR in Relation to Epithelial‐Mesenchymal Transition and Cancer Stem Cells in Molecular Subtypes of Endometrial Cancer,” International Journal of Biological Markers 31 (2016): e245‐251, 10.5301/jbm.5000187. 26868332

[19] Y. Shen , X. Wang , L. Lu , and W. Meng , “Aberrant NEAT1 Promotes Migration in Endometrial Cancer and as Marker of Poor Prognosis,” International Journal of Clinical and Experimental Pathology 10, no. 3 (2017): 3716–3721.

[20] Q. Guan , Q. Zheng , and Q. L. Ren , “Expression of Cancer⁃Associated Region Long Non⁃Coding RNA 5 in Endometrial Carcinoma and Its Value in Predicting the Prognosis,” Anhui Medical and Pharmaceutical 23 (2019): 2480–2483.

[21] L. Yang , C. F. Guo , and Y. He , “The Expression of LncRNA AFAP1⁃AS1 in Endometrial Cancer and Its Relationships With Clinicopathological Parameters and Prognosis,” Journal of Tropical Medicine 19 (2019): 1463–1467.

[22] T. Y. Wu , “Expressions of LncRNA H19,miR‐29b and miR‐124 in Endometrial Carcinoma and Their Clinical Significance,” Laboratory Medicine and Clinic 16, no. 11 (2019): 1483–1487.

[23] B. Mai , Y. X. Chen , G. Y. Hu , X. P. Luo , and T. Y. Liu , “Long Non⁃Coding RNA LINC00393 in Endometrial Cancer and Its Prognostic Im⁃ Plications,” Journal of Molecular Diagnositics 11 (2019): 198–203.

[24] T. T. Wang and F. Wang , “Expression and Clinical Significance of lncRNA ZFAS1 and miR‐590‐3p in Endometrial Carcinoma,” Chinese Journal of Birth Health & Heredity 28 (2020): 1183–1186, 10.13404/j.cnki.cjbhh.2020.10.006.

[25] X. Zhao , Y. Fan , C. Lu , et al., “PCAT1 Is a Poor Prognostic Factor in Endometrial Carcinoma and Associated With Cancer Cell Proliferation, Migration and Invasion,” Bosnian Journal of Basic Medical Sciences 19 (2019): 274–281, 10.17305/bjbms.2019.4096. 31136293 PMC6716094

[26] S. Wang , “Expression and Clinical Significance of lncRNA AFAP1‐AS in Endometrial Carcinoma,” Hebei Medical Journal 42 (2020): 2046–2049.

[27] L. Shan , T. Zhao , and Y. Wang , “Upregulation of Serum lncRNA DLEU1 Predicts Progression of Premalignant Endometrial Lesion and Unfavorable Clinical Outcome of Endometrial Cancer,” Technology in Cancer Research & Treatment 19 (2020): 1533033820965589, 10.1177/1533033820965589. 33327893 PMC7750898

[28] Y. Yang , J. Wu , H. Zhou , W. Liu , J. Wang , and Q. Zhang , “STAT1‐Induced Upregulation of lncRNA LINC01123 Predicts Poor Prognosis and Promotes the Progression of Endometrial Cancer Through miR‐516b/KIF4A,” Cell Cycle 19 (2020): 1502–1516, 10.1080/15384101.2020.1757936. 32401659 PMC7469438

[29] Y. Sun , X. Gao , P. Li , L. Song , and L. Shi , “LncRNA ZFAS1, as a Poor Prognostic Indicator, Promotes Cell Proliferation and Epithelial‐Mesenchymal Transition in Endometrial Carcinoma,” Personalized Medicine 18 (2021): 43–53, 10.2217/pme-2020-0014. 33151128

[30] X. Yin , X. Li , G. Feng , Y. Qu , and H. Wang , “LINC00565 Enhances Proliferative Ability in Endometrial Carcinoma by Downregulating KLF9,” Oncotargets and Therapy 13 (2020): 6181–6189, 10.2147/OTT.S249133. 32636642 PMC7334012

[31] J. Q. Ke , Z. Shen , and W. P. Hu , “LncRNA DCST1‐AS1 Was Upregulated in Endometrial Carcinoma and May Sponge miR‐92a‐3p to Upregulate Notch1,” Cancer Management and Research 12, no. 18 (2020): 1221–1227, 10.2147/CMAR.S234891. 32110096 PMC7035894

[32] W. Gf and W. Ln , “LncRNA SNHG14 Promotes Proliferation of Endometrial Cancer Through Regulating microRNA‐655‐3p,” European Review for Medical and Pharmacological Sciences 24, no. 20 (2020): 10410–10418, 10.26355/eurrev_202010_23391. 33155197

[33] Q. Y. Li , J. Q. Shen , J. H. Li , D. F. Dai , M. Saeed , and C. X. Li , “LINC00958/miR‐3174/PHF6 Axis Is Responsible for Triggering Proliferation, Migration and Invasion of Endometrial Cancer,” European Review for Medical and Pharmacological Sciences 25 (2021): 6853–6861, 10.26355/eurrev_202111_27233. 34859848

[34] J. Yu , Q. Fan , and L. Li , “The MCM3AP‐AS1/miR‐126/VEGF Axis Regulates Cancer Cell Invasion and Migration in Endometrioid Carcinoma,” World Journal of Surgical Oncology 19 (2021): 213, 10.1186/s12957-021-02316-0. 34256796 PMC8278665

[35] Z. Y. Gong , “Expression and Clinical Significance of Long Non‐Coding RNA Actin Fibrillin‐Associated Protein 1‐Antisense RNA in Endometrial Cancer,” Maternal and Child Health Care of China 37, no. 4 (2022): 713–716.

[36] Y. G. Ma , Y. Zan , and M. Wang , “Expression and Clinical Significance of LncRNA NNT‐AS1 and miR‐424 in Endometrial Carcinoma,” International Journal of Laboratory Medicine 42 (2021): 1376–1381.

[37] T. T. Wang and F. Wang , “Expressions and Clinical Significances of LncRNA CASC9 and MiR‐193a‐5p in Endometrial Carcinoma,” Labeled Immunoassays and Clinical Medicine 1 (2021): 65–70.

[38] J. Y. Liu , H. L. Zhang , B. P. Li , C. Li , and C. Y. Guo , “Expression of Long Non‐Coding RNA SNHG1 in Endometrial Carcinoma and Its Regulation of PI3K/AKT Signaling Pathway,” Journal of Modern Laboratory Medicine 37 (2022): 119–124.

[39] Y. J. Guo , N. N. Zhao , and J. L. Zhou , “Clinical Value of Long Chain Noncoding RNA‐LINP1 in Evaluating the Prognosis of Patients With Endometrial Cancer,” Clinical Medicine of China 12 (2021): 426–430.

[40] D. Liu , M. Qiu , L. Jiang , and K. Liu , “Long Noncoding RNA HOXB‐AS1 Is Upregulated in Endometrial Carcinoma and Sponged miR‐149‐3p to Upregulate Wnt10b,” Technology in Cancer Research & Treatment 19 (2020): 153303382096746, 10.1177/1533033820967462. PMC7592328 33073693

[41] X. L. Zhu and S. M. Liu , “Expression Level of lncRNA SNHG1 in Endometrial Cancer Tissues and Its Clinical Significance,” Chinese Journal of Cancer Biotherapy 27 (2020): 1118–1125.

[42] Y. Shen and C. Y. Xiao , “The Relationship Between Long Non‐Coding RNA Overexpression and Clinicopathological Features and Prognosis in Endometrial Cancer: A Meta‐Analysis,” Journal of International obstetrics and Gynecology 45 (2018): 643–647.

[43] L. Yan , J. Zhou , Y. Gao , et al., “Regulation of Tumor Cell Migration and Invasion by the H19/Let‐7 Axis Is Antagonized by Metformin‐Induced DNA Methylation,” Oncogene 34 (2015): 3076–3084, 10.1038/onc.2014.236. 25088204

[44] J. T. Fan , Z. Y. Zhou , Y. L. Luo , et al., “Exosomal lncRNA NEAT1 From Cancer‐Associated Fibroblasts Facilitates Endometrial Cancer Progression via miR‐26a/b‐5p‐Mediated STAT3/YKL‐40 Signaling Pathway,” Neoplasia 23 (2021): 692–703, 10.1016/j.neo.2021.05.004. 34153644 PMC8233173

[45] X. L. Li and L. J. Hu , “Research on Expression and Clinical Characteristics of Long‐Chain Non‐Coding RNA ZEB1‐AS1 in Endometrial Carcinoma and Resistance to Chemotherapy Drugs,” Journal of Modern Laboratory Medicine 34 (2019): 35–39.

[46] M. Wang , L. Fu , Y. Xu , S. Ma , X. Zhang , and L. Zheng , “A Comprehensive Overview of Exosome lncRNAs: Emerging Biomarkers and Potential Therapeutics in Gynecological Cancers,” Frontiers in Oncology 13 (2023): 1138142, 10.3389/fonc.2023.1138142. 37007117 PMC10063919

[47] J. Verma , B. J. Monk , and A. H. Wolfson , “New Strategies for Multimodality Therapy in Treating Locally Advanced Cervix Cancer,” Seminars in Radiation Oncology 26 (2016): 344–348, 10.1016/j.semradonc.2016.05.003. 27619255

